# Phosphorus and magnesium interactively modulate the elongation and directional growth of primary roots in *Arabidopsis thaliana* (L.) Heynh

**DOI:** 10.1093/jxb/erv181

**Published:** 2015-04-28

**Authors:** Yaofang Niu, Gulei Jin, Xin Li, Caixian Tang, Yongsong Zhang, Yongchao Liang, Jingquan Yu

**Affiliations:** ^1^Department of Horticulture, College of Agricultural and Biotechnology, Zhejiang University, Hangzhou 310058, PR China; ^2^College of Environmental and Resource Sciences, Zhejiang University, Hangzhou 310058, PR China; ^3^College of Agricultural and Biotechnology, Zhejiang University, Hangzhou 310058, PR China; ^4^Tea Research Institute, Chinese Academy of Agricultural Science, Hangzhou, 310008, PR China; ^5^Centre for AgriBioscience, La Trobe University, Melbourne Campus, Victoria 3086, Australia

**Keywords:** Auxin transport, interaction, magnesium, nutrient balance, phosphorus, root growth.

## Abstract

A balanced supply of essential nutrients is an important factor influencing root architecture in many plants, yet data related to the interactive effects of two nutrients on root growth are limited. Here, we investigated the interactive effect between phosphorus (P) and magnesium (Mg) on root growth of *Arabidopsis* grown in pH-buffered agar medium at different P and Mg levels. The results showed that elongation and deviation of primary roots were directly correlated with the amount of P added to the medium but could be modified by the Mg level, which was related to the root meristem activity and stem-cell division. High P enhanced while low P decreased the tip-focused fluorescence signal of auxin biosynthesis, transport, and redistribution during elongation of primary roots; these effects were greater under low Mg than under high Mg. The altered root growth in response to P and Mg supply was correlated with AUX1, PIN2, and PIN3 mRNA abundance and expression and the accumulation of the protein. Application of either auxin influx inhibitor or efflux inhibitor inhibited the elongation and increased the deviation angle of primary roots, and decreased auxin level in root tips. Furthermore, the auxin-transport mutants *aux1-22* and *eir1-1* displayed reduced root growth and increased the deviation angle. Our data suggest a profound effect of the combined supply of P and Mg on the development of root morphology in *Arabidopsis* through auxin signals that modulate the elongation and directional growth of primary root and the expression of root differentiation and development genes.

## Highlight

Phosphorus and magnesium interactively affect root elongation and skewing by a pathway that is largely dependent upon the signalling function of auxin, which leads to accelerated cell expansion and division.

## Introduction

Nutrient interactions are important for the manifestation of deﬁciency and toxicity symptoms in many plants (Marschner, 1995). Interactions between nutrients in higher plants occur when the supply of one nutrient influences the absorption, distribution, or function of another nutrient and corresponding modified plant growth responses (Fageria, 2001). These can be positive (synergistic) or negative (antagonistic). Understanding nutrient interactions in plants is important for a balanced supply of nutrients and consequently improvement of the yield and quality of crops (Fageria, 2001).

Phosphorus (P) and magnesium (Mg) are two critical macronutrients that are required for many functions in plants, including energy generation, nucleic acid metabolism, photosynthesis, enzyme activation/inactivation, redox reactions, signalling, carbohydrate metabolism, and nitrogen fixation (Igamberdiev and Kleczkowski, 2001; Abel *et al.*, 2002; Cowan 2002; Vance *et al.*, 2003; Hörtensteiner 2009). Indeed, interactions between P and Mg are expected, as Mg is an activator of several kinase enzymes and activates most reactions involving phosphate transfer (Fageria, 2001). Skinner *et al.* (1988) reported that low P led to Mg deficiency in grapevines grown in low-pH soils; the grapevines exhibited leaf symptoms that were similar to both P and Mg deficiencies. Tissue analyses confirmed that leaves with such symptoms had less than half of the normal amounts of P and Mg (Gärtel, 1965). In addition, the translocation of Mg from roots to shoots of grapevines depends on P supply to the roots (Skinner and Matthews, 1990). The supply of P was necessary to prevent Mg deficiency in vines on Mg-sufficient but P-deficient soils in the field (Skinner and Matthews, 1990). However, the nature of such interactions is not fully understood. In particular, there is no direct evidence of interactions between P and Mg affecting root formation and growth.

Root growth and distribution in soil profiles have always been reported to be plastic in response to nutrient availability and heterogeneity in the soil (Lynch, 1995, 2007; Hell and Hillebrand, 2001; Ward *et al.*, 2008; Niu *et al.*, 2013a). Under normal conditions, the primary root, after germination, exhibits gravitropism by growing downwards. These roots are positioned downwards so as to maximize the uptake of water and nutrients (Singh *et al.*, 2014). Root tips provide the most easily accessible group of stem cells in the plant body and have been used extensively to visualize the dynamics of cell division and elongation (Casamitjana-Martínez *et al.*, 2003; Dello Ioio *et al.*, 2007). In *Arabidopsis thaliana*, root growth is maintained by the root meristem, in which the stem-cell niche, comprising the mitotically inactive quiescent centre (QC) and its surrounding stem cells, provides the source of cells for all tissues in roots. The auxin/*PLETHORA* (*PLT*) and *SCR/SHR* pathways play a crucial role in the specification and maintenance of the root stem-cell niche (Sabatini *et al.*, 2003; Dinneny and Benfey, 2008; Kornet and Scheres, 2009). *SHORT-ROOT* (*SHR*) and *SCARECROW* (*SCR*) transcription factors regulate root patterning by controlling asymmetric division in the immediate progeny of root stem cells, known as cortex/endodermis initial cells (Pysh *et al.*, 1999).

Auxin signalling and redistribution are the most widely studied phenomena in response to multiple developmental processes, including root patterning (Sabatini *et al.*, 1999; Friml *et al.*, 2002; Petersson *et al.*, 2009), cell division and cell elongation in roots (Ding and Friml, 2010), and root directional growth (Philosoph-Hadas *et al.*, 2005; Rakusová *et al.*, 2011). Polar auxin transport, which is mediated by *AUXIN RESISTANT 1* (*AUX1*) influx carriers and *PIN-FORMED* (*PIN*) efflux carriers, is essential for creating auxin gradients and for proper development of organs (Tanaka *et al.*, 2006). The impaired auxin responses in mutants of auxin influx *AUX1* (Marchant *et al.*, 1999; White, 2002; Swarup *et al.*, 2005) and PIN efflux facilitators *PIN2* (Sukumar *et al.*, 2009; Rahman *et al.*, 2010) and *PIN3* (Friml *et al.*, 2002) further emphasize the requirement for plant auxin transport in root growth.

This study used *Arabidopsis* as a model plant to examine the effect of external P and Mg supply on root growth by comparing morphological, physiological, and molecular changes, and demonstrated that auxin accumulation and redistribution might be the critical signal that controls root growth under various combinations of P and Mg supply.

## Materials and methods

### Plant material

Plants of wild-type *A. thaliana* ecotype Columbia (Col-0) were grown as a reference plant. The transgenic and mutant lines used were DR5::GFP (Bargmann and Birnbaum, 2009); PIN2::PIN2-GFP, and PIN3::PIN3-GFP provided by J. W. Pan (Zhejiang Normal University, China) (Wang *et al.*, 2013), SHR::SHR-GFP provided by Betty Kelley (Biology Department, French Science Center, Duke University, NC, NC, USA), WOX5:GFP and CyCB1; 1::GUS provided by G. H. Mi (China Agricultural University, China) (Liu *et al.*, 2013), auxin-transport mutant *aux1-22* provided by M. J. Bennett (University of Nottingham, Nottingham, UK), and *eir1-1* (a loss-of-function mutant for *PIN2*) (Roman *et al.*, 1995).

### Growth medium

The P and Mg basal medium (CMgCP), which was used as a control, contained the following (µM): 1500 KNO_3_, 500 NaH_2_PO_4_, 1000 CaCl_2_, 250 (NH_4_)_2_SO_4_, 1000 MgSO_4_, 1250 Na_2_SO_4_, 25 Fe-EDTA, 10 H_3_BO_3_, 0.5 MnSO_4_, 0.5 ZnSO_4_, 0.1 CuSO_4_, and 0.1 (NH_4_)_6_Mo_7_O_24_ (Hoagland and Arnon, 1938). Various P and Mg treatments were achieved by altering the concentrations of NaH_2_PO_4_ and MgSO_4_ in the basal medium. Thus, the low-Mg and low-P (LMgLP) medium contained 1 µM MgSO_4_ and 0.5 µM NaH_2_PO_4_, the high-Mg and low-P (HMgLP) medium contained 10 000 µM MgSO_4_ and 0.5 µM NaH_2_PO_4_, the low-Mg and high-P (LMgHP) medium contained 1 µM MgSO_4_ and 3000 µM NaH_2_PO_4_, and the high-Mg and high-P (HMgHP) medium contained 10 000 µM MgSO_4_ and 3000 µM NaH_2_PO_4_. Notably, more than these Mg and P concentrations were used, as shown in Supplementary Fig. S1 available at *JXB* online. The accurate nutrient solution composition of the phosphate and Mg addition solutions is shown in Supplementary Table. S1 available at *JXB* online. Meanwhile, the medium with lower concentrations of P was made by substituting sodium sulfate for sodium dihydrogen phosphate so that the level of Na^+^ in the medium remained at 3000 µM and decreasing the difference in SO_4_^2–^ concentration among the treatments. Concentrations of Mg in the medium were adjusted by manipulating the concentration of MgSO_4_. Although small differences were present among the P and Mg treatments, such differences in SO_4_^2–^ ion had little effect on root morphogenesis of *Arabidopsis* (Gruber *et al.*, 2013; Niu *et al.*, 2014). Importantly, as shown in Supplementary Fig. S2 available, we also observed the root growth of wild-type *Arabidopsis* grown in P and Mg media that were formulated by replacing MgSO_4_ with MgCl_2_.

The medium pH was buffered with 0.5% 2-(-*N*-morpholino)ethanesulfonic acid (MES) at pH 5.8 to minimize the pH change before autoclaving. The concentrations of P and Mg in the control were 500 and 1000 µM, respectively, which have been adopted for *Arabidopsis* growth by many plant biologists (e.g. Lanquar *et al.*, 2009; Møller *et al.*, 2009). In addition, our preliminary experiment showed that 3000 µM NaH_2_PO_4_ and 10 000 µM MgSO_4_ did not cause any toxicity symptoms during the experimental period. For the above reasons, five representative treatments of LMgLP, HMgLP, CMgCP, LMgHP, and HMgHP were chosen to study the interactions between and P and Mg on the elongation and growth direction of primary roots of *Arabidopsis*.

### Growth condition

*Arabidopsis* seeds were surface sterilized for 5min in 75% alcohol and washed three times with sterile water. The seeds were then placed on ‘P-Mg’ agar medium containing 1.2 % (w/v) sucrose 0.8% (w/v) agar in 10×10cm^2^ plates with a grid schematic engraved below the plate. Plates were positioned in racks and oriented in a vertical position, and were kept at 4 °C for 48h in the dark for seed stratification. Thereafter, the plates were transferred to a growth chamber under a 10h light/14h dark photoperiod at a constant temperature of 22 °C, 60% relative humidity, and light intensity of 120 µmol photons m^−2^ s^−1^ as described by Niu *et al.* (2013b). Six seedlings were grown in each plate and treatments were replicated at least three times.

### Drug treatments

To study the inhibitory effect of auxin on root elongation and directional growth, *Arabidopsis* seedlings were exposed to different P and Mg treatments supplied with 50 µM 1-naphthoxyacetic acid (1-NOA, an auxin influx inhibitor) or 10 µM *N*-1-naphthylphthalamic acid (NPA, an auxin efflux inhibitor). For the simultaneous treatment, the plants were grown for 7 days on agar medium containing 1-NOA or NPA as follows: (i) 50 µM 1-NOA+LMgLP; (ii) 50 µM 1-NOA+HMgLP; (iii) 50 µM 1-NOA+CMgCP; (iv) 50 µM 1-NOA+LMgHP; (v) 50 µM 1-NOA+HMgHP; (vi) 10 µM NPA+LMgLP; (vii) 10 µM NPA+HMgLP; (viii) 10 µM NPA+CMgCP; (ix) 10 µM NPA+LMgHP; and (x) 10 µM NPA+HMgHP.

### Length and deviation of primary roots

Seedlings were photographed with a high-resolution digital camera (Sony RX100, Japan) after 7 or 14 d of treatment. All pictures displayed in our study were photographed from the back of the plate. Photographs were analysed and quantified for root length using the public domain image analysis program ImageJ version 1.43 (http://rsb.info.nih.gov/ij/). The scale was set for the picture within the program. Digital images were captured and processed using ImageJ. The root deviation was measured by calculating the angle of roots from the vertical axis using a protractor. Seedlings of less than 14-d old with straight roots were used for most experiments. For the split-plate experiment, *Arabidopsis* seeds were exposed to a split-plate such that the top/bottom half of the plate contained LMgLP/HMgLP, HMgLP/LMgLP, HMgHP/HMgLP, HMgLP/HMgHP, LMgHP/HMgHP, or HMgHP/LMgHP medium.

The meristematic zone was defined as the region of isodiametric cortical cells from the QC up to the cortical cell that was twice the length of the immediately preceding cell, according to Takatsuka and Umeda (2014). For interaction-response assays, the average distance from the QC to each individual cell was computed as a function of the cell number. Cells were counterstained with propidium iodide (PI) at 10 μg ml^–1^.

### Analysis of P and Mg in plant tissue

After 7 d of growth at various P and Mg concentrations, the plants were harvested, washed thoroughly with deionized water, separated into shoots and roots, and dried in an oven at 75 °C for 12h. The samples were then weighed, digested in sulfuric acid/hydrogen peroxide, and analysed for total P concentration using the vanadium/molybdenum blue spectrometric method (Niu *et al.*, 2013b). ‘P concentration in roots’ was calculated by total amount of P per root/root dry weight. For Mg analysis, the dried root and shoot samples were wet digested in concentrated HNO_3_/H_2_O_2_ at 90, 120, and 140 °C for 2h each until there was no brown fume, and then further digested at 180 °C until the digest became clear. Concentrations of Mg in the digests were analysed by inductively coupled plasma mass spectrometry (7500 Series ICP-MS systems Agilent, USA), and were calculated on the basis of dry weight of the root.

### Histochemical localization of β-glucuronidase (GUS) expression

The histochemical analysis of GUS activity was examined as described previously (Raghothama *et al.*, 1997). The samples were submersed in GUS reaction mix [0.05 mm sodium phosphate buffer, pH 7.0, 1mm X-Gluc and 0.1% (v/v) Triton X-100], vacuum dried, and then incubated at 37 °C for 6h. Green tissues were destained with ethanol prior to observation. The stained tissues were visualized and images acquired using an Eclipse E600 microscope (Nikon, Melville, NY, USA) and recorded using a Penguin 150CL cooled CCD camera (Pixera, Los Gatos, CA, USA).

### Laser-scanning confocal microscopy

After 7 d of growth at various P and Mg concentrations, these transgenic plants were harvested, mounted, and photographed with a Zeiss LSM 780 (Carl Zeiss MicroImaging GmbH, Germany). Roots were stained in 10 μg ml^–1^ of PI for 2–3min, rinsed in ddH_2_O, and green fluorescent protein (GFP) was excited with a 488nm laser line and detected at 500–530 nm, while PI was excited with 561nm laser line and detected at 570–630 nm. For the yellow fluorescent protein (YFP)-tagged reporters, the excitation wavelengths were 514nm, and fluorescence was collected in the range of 520–540nm (rendered in yellow). Roots were placed on the stage of a Zeiss Axiovert inverted microscope attached to a LSM780 laser-scanning confocal microscope and imaged using a Zeiss 780 confocal microscope equipped with a ×10 and ×20 1.4 numerical aperture Plan-Apochromat water objective lens as described by Niu *et al.* (2014). Each frame represents a 7 s scan of the laser. Finally, images were processed and exported with ZEN 2011 software (Carl Zeiss, Jena, Germany). The signal intensities of green fluorescence in images were quantified using ImageJ version 1.43.

### Extraction of total RNA and quantitative PCR

Total RNA was extracted from about 50mg of fresh root tissues using RNAisoPlus (Takara, Otsu, Shiga, Japan). Four independent biological replicates were performed on independent root material from different plants. All RNA samples were checked for DNA contamination before cDNA synthesis (Niu *et al.* 2011). The mRNA levels of all genes were detected using a SYBR Green RT-PCR kit (Takara, Otsu, Shiga, Japan) with pairs of gene-specific primers (Table 1). The real-time PCR analysis was performed with a Mastercycler ep *realplex* (Eppendorf, California, USA). *UBQ10* was chosen as the housekeeping reference gene.

**Table 1.** GenBank accession numbers of genes and their primer and sequence information for quantitative PCR used in this study

GenBank accession no.	Gene name	Forward primer (5′→3′)	Reverse primer (5′→3′)
AT5G20730.1	*ARF7*	AGTGGCGGAATCTTCAGATTGGA	AAAAGCGAGGTCGGAAAAATGGAG
AT2G38120.1	*AUX1*	AGCTGCGCATCTAACCAAGTG	GATGAGATAAGCAGTCCAGCTTCC
AT5G01240.1	*LAX1*	TATGAAGAGCTTCCTCTGGCATGG	ACAGCACTTGTGCCACTTGATTTG
AT2G21050.1	*LAX2*	TCGGTGGACATGCTGTTACTGTAG	GCACGTAGAGTGTTGCAAACAGG
AT1G77690.1	*LAX3*	TGTGAAACACTCTGGTCCAACCAC	CCACATAGCGTGCATTATCTCCAC
AT3G02260.1	*LPR1*	CATCCAGCAGGTGTGTTCTTGTCC	TGTTTGCTTTGGGAAAGGAACCTG
AT5G57090.1	*PIN2*	TCACGACAACCTCGCTACTAAAGC	GTCTTGGTCCATTTCCACATGCC
AT1G70940.1	*PIN3*	GAGCACCTGACAACGATCAAGG	CTTGCTGGATGAGCTACAGCTTTG
AT3G20840.1	*PLT1*	ATCGTGGTGTCACAAGACATCG	TTCCTAGACTGGCCTTCCCTTC
AT1G51190.1	*PLT2*	AAGATGGCAAGCAAGGATCGG	GCTTCTTCCTCCGTGCTGAATG
AT3G54220.1	*SCR*	TCTTTGACTCACTGGGAGCAAGC	TGCTGTTCCACGACATGTCTCTC
AT4G37650.1	*SHR*	TAGGGTTTGCTTCGAGTCATGGG	TGCACGCTCTAGCATCAACCTC
AT3G62980.1	*TIR1*	ATCGCTGCCACTTGCAGGAATC	GGCCACTAACGTCGTCAACATCAC

### Statistical analysis

All statistical analyses were conducted with DPS software (Stirling Technologies, China). Means were compared by *t*-test or Fisher’s least significant difference test at *P*<0.05 in all cases.

## Results

### Interactive effect of P and Mg on root growth and deviation

**Fig. 1.**
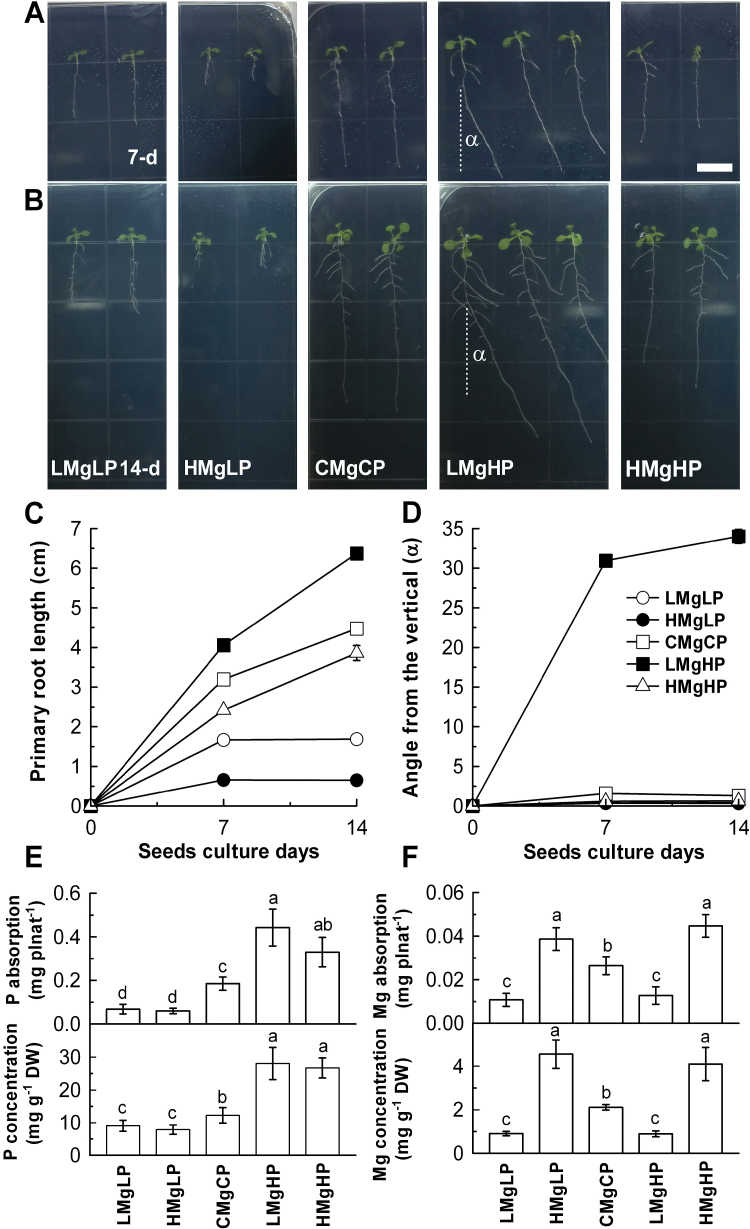
(A–D) Elongation and directional growth at days 7 and 14. (E, F) Total content and root concentrations of P (E) and Mg (F) at day 14 in roots of wild-type *Arabidopsis* seedlings grown vertically in agar medium with different concentrations of P and Mg. LMgLP, low-Mg and low-P medium containing 1 µM Mg^2+^ and 0.5 µM H_2_PO_4_^–^; HMgLP, high-Mg and low-P medium containing 10 000 µM Mg^2+^ and 0.5 µM H_2_PO_4_^–^; CMgCP, control treatment containing 1000 µM Mg^2+^ and 500 µM H_2_PO_4_^–^; LMgHP, low-Mg and high-P medium containing 1 µM Mg^2+^ and 3000 µM H_2_PO_4_^–^; HMgHP, high-Mg and high-P medium containing 10 000 µM Mg^2+^ and 3000 µM H_2_PO_4_^–^. The degree of the primary root deviation angle from the vertical gravity vector (α) were measured using a protractor. Data are means±SD (*n*=10). Means with the same letter within a root segment in (E) and (F) were not significantly different at *P*≤0.05. Bar, 1cm. (This figure is available in colour at *JXB* online.)

We first compared the effects of P and Mg level on the root growth at three P levels and three Mg levels with different combinations. The lengths of primary roots at both 7 and 14 d increased with the increase in P level but decreased with the increase in Mg level in the growth medium (Supplementary Fig. S1). Importantly, replacement of MgSO_4_ with MgCl_2_ in P and Mg treatment led to the root phenotypes shown in Fig. S1, which was attributed mainly to the interactive effect of the P and Mg ions but not sulfate or both combined Mg and sulfate (Supplementary Fig. S2). We then grew the plants with low P (0.5 µM, LP) or high P (3000 µM, HP) with low Mg (1 µM, LMg) or high Mg (10 000 µM, HMg) levels, together with normal P (500 µM, CP) and Mg (1000 µM, CMg). The lengths of the primary roots were 47 and 76% lower in the LMgLP and HMgLP treatments, respectively, than in the CMgCP treatment at 7 d, compared with 62 and 85% at 14 d. The lengths of primary roots grown at HMgHP also decreased by 24 and 13% at 7 and 14 d, respectively, compared with the control (Fig. 1A–C). In contrast, high P increased the elongation of primary roots by 42 and 14% at 7 and 14 d, respectively, when the Mg supply decreased from 1000 to 1 μM (Fig. 1C). It appears that the elongation rate of primary roots decreased over time in the LMgLP and HMgLP treatments but increased in the LMgHP and HMgHP medium, compared with the control. The shortest primary roots developed at HMgLP (10 000 µM Mg and 0.5 µM P), while the longest roots were observed at LMgHP (1 µM Mg and 3000 µM) (Fig. 1A, B). Notably, *Arabidopsis* (Col-0) primary roots growing vertically on agar plates showed a tendency to deviate from a strict gravitational direction with LMgHP treatment and this was always to the left (if observed from the lid of the plate) in repeated experiments (Fig. 1A, D).

To check if this root directional response was a direct effect of gravistimulation, wild-type seedlings were grown for 7 d in different P and Mg media and then rotated 90 º for 24h. Interestingly, when rotated 90 º for 24h, the roots gravibended in the elongation zones, while the roots grown at LMgHP also exhibited deviation from straight/vertical growth (Supplementary Fig. S3 available at *JXB* online), suggesting that the root directional response to low Mg and high P was unlikely to have resulted from hampered stimulation of gravity.

The concentration of P in roots and total P content per plant of wild-type *Arabidopsis* increased progressively with an increasing supply of P to the medium (Fig. 1E). The same was true for Mg (Fig. 1F). Although P concentration and total P content in plants were similar in the LMgLP and HMgLP treatments, and at LMgHP and HMgHP, the length of the primary root was shorter with a high-Mg supply than with a low-Mg supply (Fig. 1). Notably, a high supply of Mg consistently tended to reduce the P concentration to levels below those measured under low Mg. It is plausible that the inhibition of primary root growth under high Mg resulted from Mg toxicity in the roots, especially when low P was supplied. To test this possibility, six split-plate experiments were performed. Regardless of the high-Mg supply in either the top or bottom of the plate, a low-P supply consistently decreased root length as observed in the HMgLP treatment (Supplementary Fig. S4A, B at *JXB* online). Interestingly, under a high-Mg supply, only a high-P supply in the lower part of the plate increased root length (Supplementary Fig. S4C, D), indicating that a localized supply of high P strongly enhanced root growth under a high-Mg supply. Additionally, at high P, a low-Mg supply in either the lower or upper part of the plate decreased root growth but did not change root growth direction, whereas a localized supply of high Mg increased root growth compared with the uniform supply of LMgHP and HMgHP, respectively (Supplementary Fig. S4E, F).

### Root meristem activity, cell production, and the root stem-cell niche

**Fig. 2.**
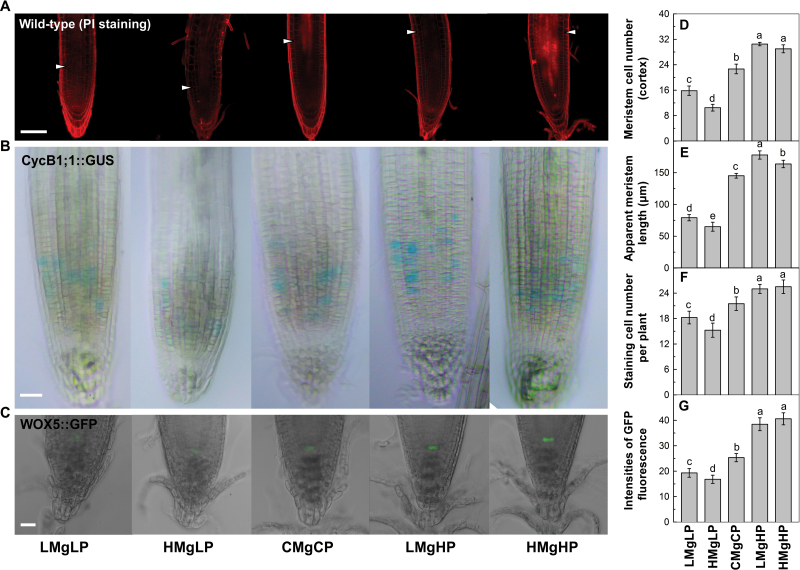
Interactive effect of P and Mg supply on apparent root meristem size, the number of dividing cells, and CycB1;1::GUS and WOX5::GFP expression. Confocal fluorescence micrographs of PI-stained primary root tips (A), cell-cycle reporter CycB1;1::GUS (B), QC-specific marker WOX5::GFP (C), the number of meristematic cortex cells (per file) (D), apparent root meristem length (E), the number of dividing cells as determined by counting the blue-green puncta in CycB1;1::GUS-stained root tips (F), and quantiﬁcation of ﬂuorescence by image analysis of confocal sections (G) of *Arabidopsis* seedlings grown for 7 d in the same treatments as in Fig. 1. Images are representative of 10–15 plants in three replicate experiments. QC cells were visualized by the WOX5::GFP marker. Expression of the WOX5::GFP marker confirmed that it was the QC cells that divided. Cells were counterstained with PI. Arrowheads in (A) indicate the approximate position where cells begin to elongate noticeably. Means followed by a same letter within a root segment in (D)–(G) are not significantly different at *P*≤0.05. Bars, 50 µm (A–C).

Cell production depends on the number of dividing cells (i.e. the length of root meristem) and the rate of cell division (Baskin, 2013), while the root meristem is the distance between the QC and the noticeably elongated cortical cells, which represents the zone of root cell division (Dello Ioio *et al.*, 2007; Baskin, 2013, Takatsuka and Umeda, 2014). By using PI staining, we found that the number of cortical cells in the meristem was 32 and 51% lower in the LMgLP and HMgLP treatments than at CMgCP, respectively, at 7 d (Fig. 2A, D). Likewise, the length of the meristem treated for 7 d at LMgLP and HMgLP decreased by 37 and 48%, respectively (Fig. 2E). By comparison, the number of cortical cells in the meristem was 33 and 26% higher, and the length of meristems 27 and 23 % greater in LMgHP and HMgHP treatments versus the control, respectively (Fig. 2D, E). As cell extension is another possible cause of primary root elongation, we examined the cell length in the elongating and maturation zones of the root. We observed that cell length was significantly shorter in the low-P treatments but longer in the high-P treatments, compared with the control (Supplementary Fig. S5 available at *JXB* online), indicating that high P promotes cell elongation in addition to cell division. The deviation from vertical growth at LMgHP should result from unequal cell expansion in both meristem and elongation zones.

The interactive effect of P and Mg on cell division was assessed based on expression of the mitotic cyclin *CycB1;1* (which marks the G_2_/M phase of the cell cycle; Colon-Carmona *et al.*, 1999) by employing the transcriptional and translational fusion CycB1;1::GUS. The expression of the cell-cycle marker CycB1;1::GUS was suppressed at LMgLP and HMgLP but promoted at LMgHP and HMgHP (Fig. 2B), indicating that low P decreased while high P increased the root meristematic activity of *Arabidopsis*. Furthermore, irrespective of the P supply, high Mg significantly reduced the number of cells in the M phase over time (Fig. 2F).

Root stem cells serve as the source for new derivative cells and are essential for the development of root meristems (Jiang and Feldman, 2005). By using transgenic plants harbouring the QC-specific marker of a *WUSCHEL RELATED HOMEOBOX5* (WOX5)::GFP marker gene at the centre of the stem-cell niche (Blilou *et al.*, 2005), we analysed the expression of the marker gene in response to the P and Mg treatments. Compared with CMgCP treatment, the GFP signal of WOX5::GFP was clearly observed in the QC cells and intensified in the root at high P, regardless of Mg supply (Fig. 2C). Notably, the GFP signal was significantly weakened by a low-P supply compared with the control, with suppression being greater under high Mg than under low Mg. The GFP fluorescence of 12 roots of each treatment confirmed this observation (Fig. 2G).

### Auxin accumulation and redistribution in the root tips

**Fig. 3.**
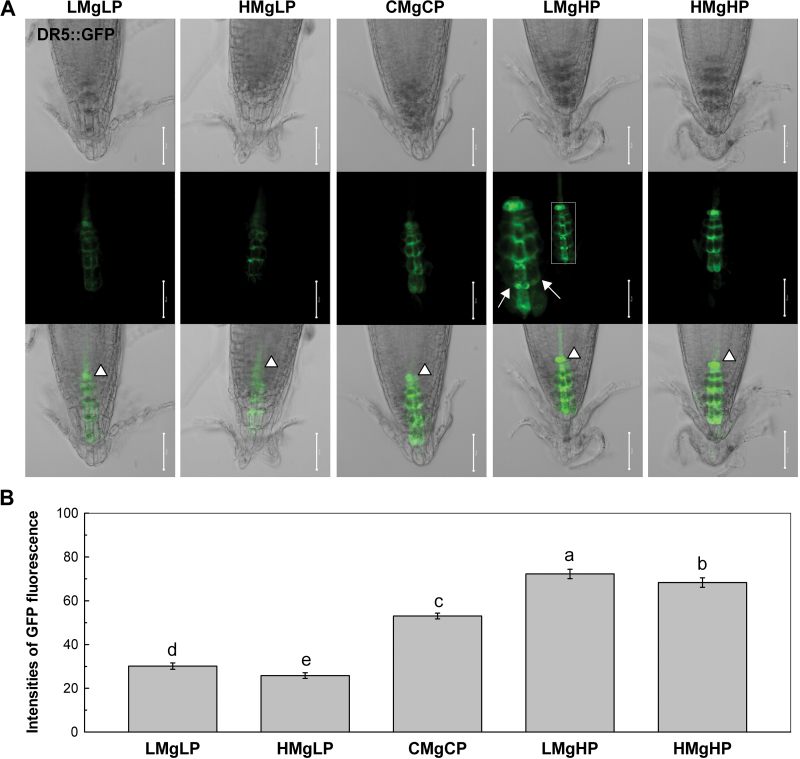
*Arabidopsis* DR5::GFP reporter lines were grown vertically for 7 d in growth medium with different concentrations of P and Mg, as in Fig. 1. (A) Confocal images of expression of the auxin-specific reporter gene DR5 as green fluorescence in the root tips. In the middle image of the fourth column in (A), a magnified view of the outlined portion is shown; arrows indicate the differentiated GFP ﬂuorescence level. In the bottom row, arrowheads indicate expression of DR5::GFP at the QC. Images are representative of 10–15 plants in three replicated experiments. Bars, 50 μm. (B) Quantiﬁcation of ﬂuorescence by image analysis of confocal sections. Data represent means±SD (*n*=12). Means with the same letter were not significantly different at *P*≤ 0.05.

To evaluate the contribution of auxin responsiveness in the altered primary root formation in response to P and Mg, we used transgenic plants expressing a DR5::GFP construct, which is useful in studying auxin-regulated gene expression in *Arabidopsis* (Ulmasov *et al.*, 1997). As shown in Fig. 3A, compared with the control CMgCP, LMgHP and HMgHP increased DR5::GFP expression in the root tip with the increase being greater at LMgHP than at HMgHP. The opposite was true for the plants grown at LMgLP and HMgLP. The GFP fluorescence of 12 roots of each treatment confirmed this observation (Fig. 3B). In particular, the root tips grown at LMgHP presented asymmetric DR5::GFP expression, with auxin accumulation at one side, which drove the differential growth and deviation (Fig. 3A).

**Fig. 4.**
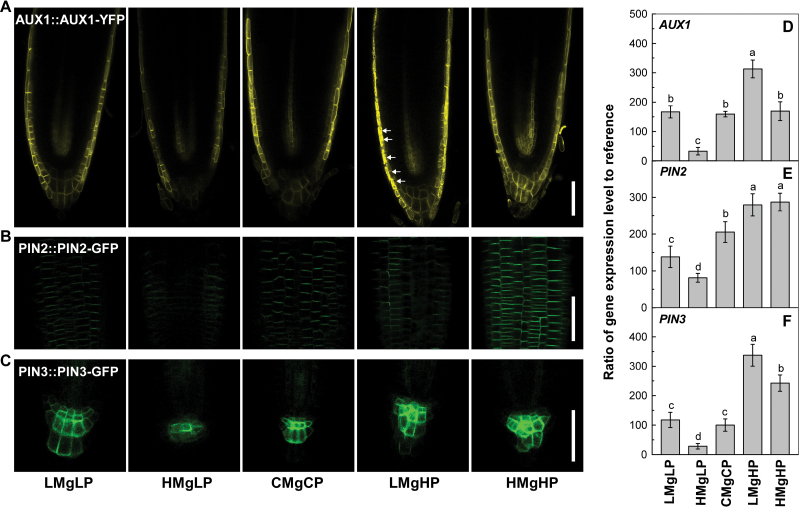
Marker lines AUX1::AUX1–YFP (A), PIN2::PIN2–GFP (B) and PIN3::PIN3–GFP (C) were grown vertically with the P and Mg treatments given in Fig. 1. YFP or GFP ﬂuorescence of the root tips was recorded by confocal microscopy. The arrows in (A) indicate AUX1::AUX1–YFP expression inside and around the epidermis. Relative expression of *AUX1* (D), *PIN2* (E), and *PIN3* (F) genes in the roots of 7-d-old wild-type seedlings was measured by quantitative PCR as described in Materials and methods. Relative gene expression (∆*C*_t_) was calculated by normalizing target gene *C*_t_ values to those of the endogenous control gene, *UBQ10*. For each gene, the mRNA level was normalized to that of *UBQ10*. Bars, 50 µm.

Well-documented reporter lines relating to auxin transport, comprising AUX1::AUX1–YFP, PIN2::PIN2–GFP, and PIN3::PIN3–GFP (Friml *et al.*, 2002, 2003; Michniewicz *et al.*, 2007; Petrášek and Friml, 2009) were used to investigate auxin redistribution at the root apex. As shown in Fig. 4, the expression of AUX1::AUX1–YFP, PIN2::PIN2–GFP, and PIN3::PIN3–GFP was strongly downregulated by HMgLP but upregulated by LMgHP and HMgHP. Importantly, AUX1::AUX1–YFP and PIN2::PIN2–GFP were expressed asymmetrically in the epidermis and cortex of the meristem (Fig. 4A, B), which would affect the auxin reflux loop and finally alter cell division and root deviation.

In addition, the expression of *AUX1*, *PIN2*, and *PIN3* at the transcriptional level was quantified at 7 d by means of quantitative real-time PCR. Fig. 4 shows that LMgHP downregulated but HMgLP upregulated the expression of *AUX1* and *PIN3*. These results indicated that the growth and directional response of primary roots to P and Mg supply were correlated with the abundance and expression of *AUX1* and *PIN2* mRNA and localization of the protein.

### Role of polar auxin transport in the response of root tips to P and Mg supply

**Fig. 5.**
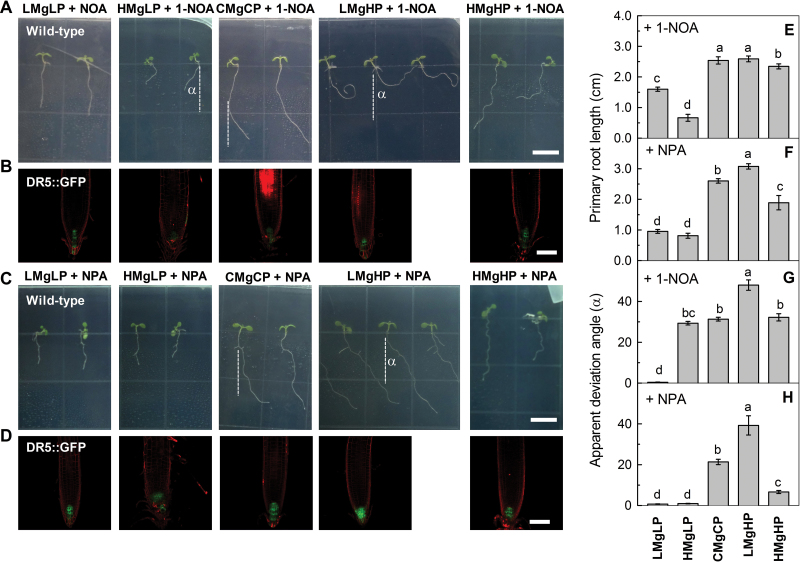
Effects of 1-NOA and NPA on elongation (A, C, E, F,) and deviation (A, C, G, H) of primary roots of *A. thaliana* wild-type seedlings and on the expression of auxin-specific reporter gene DR5 (B, D) in PI-stained primary root tips of the DR5::GFP transgenic line. The seedlings were treated for 7 d with the following: (i) 50 µM 1-NOA+LMgLP; (ii) 50 µM 1-NOA+HMgLP; (iii) 50 µM 1-NOA+CMgCP; (iv) 50 µM 1-NOA+LMgHP; (v) 50 µM 1-NOA+HMgHP; (vi) 10 µM NPA+LMgLP; (vii) 10 µM NPA+HMgLP; (viii) 10 µM NPA+CMgCP; (ix) 10 µM NPA+LMgHP; and (x) 10 µM NPA+HMgHP, as indicated. Root length was measured using the ImageJ program. Deviation angle of primary roots (G, H) from the vertical gravity vector (α) was measured using a protractor. Data are means±SD (*n*=10). Means with the same letter within a panel were not significantly different at *P*≤0.05. Bars, 1cm (A, C); 50 µm (B, D). (This figure is available in colour at *JXB* online.)

The auxin-transport inhibitors 1-NOA (influx inhibitor) and NPA (efflux inhibitor) were used to confirm the transport and signalling function of auxin in mediating P/Mg-induced development of root growth. The application of either 1-NOA or NPA intensified the root phenotype, especially the root deviation induced by LMgHP. Moreover, the addition of 1-NOA or NPA in the CMgCP treatment stimulated deviation from the straight/vertical growth of primary roots, and the opposite was true for the HMgLP and HMgHP treatments (Fig. 5).

**Fig. 6.**
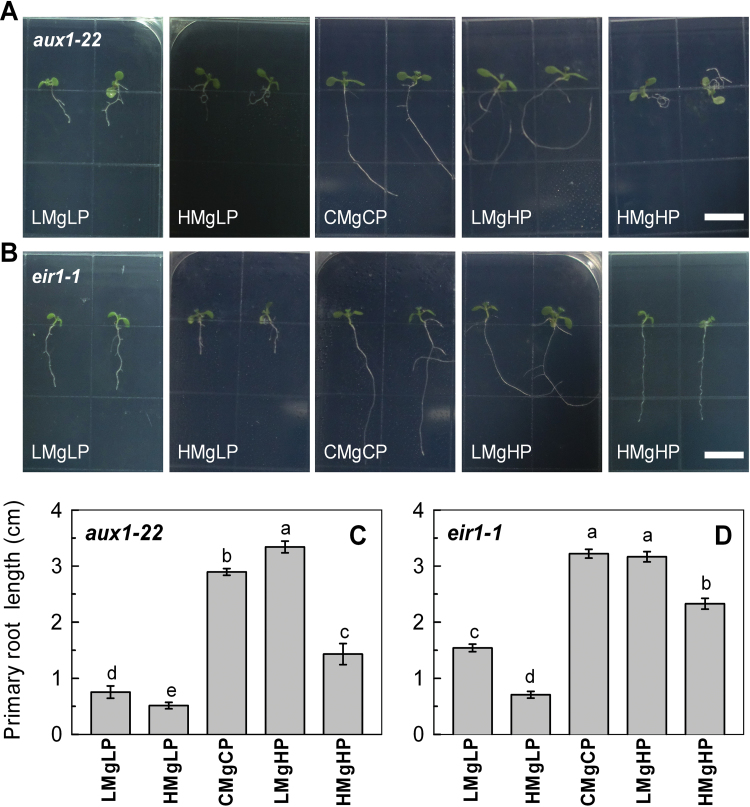
Images of root growth of *Arabidopsis* mutants *aux1-22* (A) and *eir1-1* (B) and the length of their primary roots (C, D) grown vertically in medium with P and Mg treatments as given in Fig. 1. Data are means±SD (*n*=10). Means with the same letter within a mutant were not significantly different at *P*≤0.05. Bars, 1cm. (This figure is available in colour at *JXB* online.)

The *aux1-22* and *eir1-1* (a loss-of-function mutant for PIN2) (Roman *et al.*, 1995) mutants were selected to further confirm the role of auxin transport and redistribution in regulating root growth under various P and Mg treatments. If P and Mg moderate primary root elongation and growth direction by regulating AUX1 and PIN2, then root growth of these mutants should phenocopy the P/Mg treatments and root deviation response in the mutants. Indeed, compared with the wild type, the mutants *aux1-22* and *eir1-1* displayed a greater inhibition of their primary roots at LMgLP or HMgLP and increased leftward skewing under LMgHP (Fig. 6). Interestingly, the *aux1-22* mutant plants showed severely curved roots with decreased growth when grown at HMgHP for 7 d (Fig. 6). These results suggested that P and Mg affect root elongation and growth direction by a pathway that is largely dependent on auxin transport.

### Characterization of auxin-response and root-related genes

**Fig. 7.**
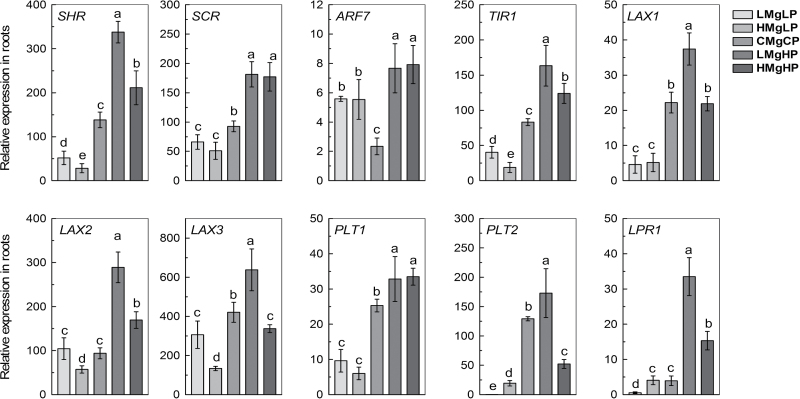
Relative expression levels of the genes *SHR*, *SCR*, *ARF7*, *TIR1*, *LAX1*, *LAX2*, *LAX3*, *PLT1*, *PLT2*, and *LPR1* in the roots of wild-type *Arabidopsis* grown for 7 d with P and Mg treatments as given in Fig. 1. Relative expression levels were calculated and normalized with respect to *UBQ10* mRNA. Data are means±SD (*n*=5). Means with the same letter within each gene were not significantly different at *P*≤0.05.

Expression of the *SHR* and *SCR* transcription factors in the *SHR/SCR* pathway plays a vital role in the specification and maintenance of the root stem-cell niche by providing a positional signal along the radial axis that differs from the longitudinal signals of the auxin pathway (Scheres, 2007; Benjamins and Scheres, 2008; Dinneny and Benfey, 2008). Compared with the normal P and Mg supply, expression of *SCR* and *SHR* was decreased by LMgLP and HMgLP treatments (Fig. 7). However, the transcript levels of both *SHR* and *SCR* were significantly increased by a high-P supply, with the increase of expression of *SHR* being greater with a high-Mg supply than a low-Mg supply (Fig. 7). In addition, compared with the control CMgCP, the accumulation of SHR was reduced by low P, as revealed by the marker lines SHR::SHR–GFP, with the reduction being greater under high Mg than under low Mg (Supplementary Fig. S6, available at *JXB* online). By comparison, the strong SHR-GFP signal was found under LMgHP and HMgHP, and the SHR protein was expressed in the LMgHP-treated root throughout the major stele (Supplementary Fig. S6). *PLETHORA 1* (*PLT1*) and *PLETHORA 2* (*PLT2*) belong to the AP2-domain transcription factor family, and are essential for determining the number of root columnar cells (Aida *et al.*, 2004). In the present study, the transcript levels of both *PLT1* and *PLT2* were significantly decreased by a low-P supply, regardless of the Mg supply (Fig. 7). In comparison, LMgHP increased expression of the *PLT1* and *PLT2* genes, whereas HMgHP increased the expression of *PLT1* but decreased that of *PLT2* compared with CMgCP. These results demonstrated that the elongation and deviation of primary roots is a phenotypic surrogate for the molecular responses to P and Mg supply of auxin/*PLETHORA* (*PLT*) and the *SCR/SHR* pathway in *Arabidopsis*.

Other selected genes are known to be involved in the auxin-mediated signalling pathway (http://www.arabidopsis.org/). For example, *AUXIN RESPONSE FACTOR7* (*ARF7*) encodes an auxin-regulated transcriptional activator and activates the expression of *IAA1* and *IAA9* in the presence of auxin. *TIR1* encodes an auxin receptor that mediates auxin-regulated transcription. *LIKE AUXIN RESISTANT1* (*LAX1*) and *LAX2* are members of the *AUX1 LAX* family of auxin influx carriers, while *LAX3* encodes an auxin influx carrier LAX3 that promotes lateral root emergence. Compared with CMgCP, LMgHP upregulated while HMgLP downregulated these genes in the roots (Fig. 7).

## Discussion

Plant roots are plastic in response to nutrient supply. When exposure to an unbalanced supply of nutrients or heterogeneous distribution in the soil, plant roots perceive changes in orientation relative to the gravity vector and initiate signal transduction events, resulting in an asymmetric redistribution of auxin, which accumulates along the bottom of the responsive tissue and encourages directional growth (Singh *et al.*, 2014). The root senses available P and Mg levels and responds accordingly by reprogramming its development and growth to shape an appropriate root system for the best utilization of nutrients in the soil profile. In this study, we showed that: (i) root elongation was increased with the increase in P level but decreased with the increase in Mg level, and low Mg with high P induced a directional growth; (ii) the combined supply of P with Mg modulated the elongation and directional growth of primary roots by affecting differentiation and elongation of cells in the root meristem and elongation zone; and (iii) P and Mg altered root growth through an auxin signalling pathway.

We found that there was a strong interaction between P and Mg to influence the root growth of *Arabidopsis* (Supplementary Fig. S1). The growth of primary roots of *Arabidopsis* was decreased by a low-Mg supply but was increased by a high-P supply in the medium. This result is consistent with the findings of López-Bucio et al. (2002), Sánchez-Calderón et al. (2005) and Jain et al. (2007) that the growth of primary roots was inhibited by low P availability as compared to the normal P supply. Furthermore, this effect of P concentration on primary root growth could be moderated by Mg supply (Supplementary Figs S1 and S2). Furthermore, the root phenotype shown in Fig. 1 was attributed mainly to the interactive effect of Mg ions with P rather than with sulfate ions (Supplementary Fig. S2). Notably, one experiment tested possible chemical reactions between 10mM MgSO_4_ and 3mM NaH_2_PO_4_ (the highest Mg and P concentrations used in this study) and showed that no precipitates were formed at pH 5.8 buffered with MES. These experiments provided evidence on the root phenotype shown in Fig. 1 resulting from physiological and molecular interactions between P and Mg but not from effects of Mg on P chemistry in the growth medium.

It is interesting to note that a supply of low Mg plus high P resulted in a growth deviation of the primary root from the vertical direction. Interestingly, with a combination of low P and low Mg, root growth was tardy and the effect of Mg deficiency became apparent after around day 14 when the primary root started to deviate from the vertical axis. This together with the result of LMgHP implied that Mg deficiency was likely to result in root skewing to the left, but this response was dependent on the seedling age and a balanced supply of Mg and P in the medium rather than the hampered stimulation of gravity (Supplementary Figs S3 and S4). It is plausible that exacerbation of the effects of low P in the presence of high Mg is due to the increase in the severity of P starvation. The results showed that there was a similar trend between ‘the total P concentration per plant’ and ‘P concentration in roots’ under P and Mg treatments. This suggests that a combination of low P and high Mg reduced P uptake and thus inhibited the root growth, or the opposite was probably true for inhibition of root growth leading to restrained P uptake. In addition, at a given level of P supply, the P concentrations in the plant were consistently lower with a supply of high rather than low Mg, although high Mg did not significantly affect P concentration in the roots. As excepted, low P with high Mg (HMgLP) in either the top or bottom part of the plate decreased root length. On the other hand, a localized supply of high P with high Mg (HMgLP/HMgHP) stimulated root growth (Supplementary Fig. S4). Based on the above findings, we can conclude that detected exacerbation of the effects of low P in the presence of high Mg is due to the increase in severity of P starvation. Root growth inhibition by low P and high Mg has been suggested as a general mechanism for stress evasion, which leads to a redirection of growth of the root system away from localized sources of stress or as a means to better exploit the soil environment towards potential novel sources of nutrients (Ruíz-Herrera and López-Bucio, 2013). A low Mg supply may lead to enhanced resistance to P deficiency.

To further clarify the cellular and molecular mechanisms of how the supply of P and Mg affected root growth, we determined the activity of cells originating from root meristems, in which most post-embryonic cell division takes place. In the present study, low P effectively decreased both the length and number of cells in the root meristems and elongating zones, and hence the reduction in the length of primary roots of the wild type was greater under high Mg than under low Mg (Fig. 2 and Supplementary Fig. S5). This response was fast (within 2 days) and required physical contact between the root tip and the low-P medium (Linkohr *et al.*, 2002; Svistoonoff *et al.*, 2007). The noticeable asymmetrical cell division and uneven rates of cell expansion in the meristem and elongation zones could explain the root growth and deviation under low Mg and high P.

Furthermore, the repression of CycB1;1::GUS under HMgLP (Fig. 2) revealed that the decreased cell number in the root meristem by low P could result from a decrease in cell division. The reduced expression of WOX5::GFP under low P confirmed that QC cells and the cells surrounding the QC might lose their function or be dead. Meanwhile, the abnormally enlarged columella cells (the cells immediately below the QC) under HMgLP treatment (Fig. 2) implied that these cells failed to execute the QC function of maintaining columella stem cells in an undifferentiated state. In contrast, high P effectively increased both cell number in the root meristem and the mitotic activity of stem daughter cells, and finally facilitated root elongation and development. Together with reports that reduced growth of the primary roots of P-deficient plants is correlated with a reduction in cell differentiation within the primary root meristem and the inhibition of cell proliferation in the root elongation zone (Sánchez-Calderón *et al.*, 2005; Svistoonoff *et al.*, 2007), our results suggest that P with Mg induced *Arabidopsis* roots to enter a determinate developmental programme in which cell division is arrested and cell differentiation is promoted.

Auxin plays important roles in the patterning of root stem cells in the root meristem (Dinneny and Benfey, 2008; Ding and Friml, 2010) and in the directional growth of *Arabidopsis* roots (Rashotte *et al.*, 2000; Band *et al.*, 2012). In this study, a high-P supply enhanced the auxin signal and maintained the maximal distribution of auxin signal marker in the QC cells. This effect of a high-P supply was greater under low Mg than under high Mg (Fig. 3). This pattern of auxin distribution leads to ectopic QC and columella specification, indicating that auxin is involved in stem-cell specification in roots (Sabatini *et al.*, 1999). This study showed that low Mg plus high P led to an asymmetric release of auxin in the auxin-sensing QC and columella cells at the root apex of *Arabidopsis* seedlings under LMgHP (Fig. 3). In the root placed vertically at LMgHP, auxin would localize in the left side, promoting inhibition of cell growth in the elongation zone. On the other hand, the low concentration of auxin on the right side of the root would provoke extension of the cells (Oliva and Dunand, 2007). Moreover, auxin was rapidly redistributed to the lower side of the root within minutes of a 90° gravity stimulus (Supplementary Fig. S2), while the asymmetry was rapidly lost as bending root tips restored partly their position to the horizontal (data no shown). This result indicated that auxin redistribution plays a role in the process of root deviation under LMgHP.

The auxin-transport carriers determine auxin accumulation and redistribution in roots. In particular, PIN2 is localized asymmetrically at epidermal cells or cortical cells of the meristem, thus creating an auxin reflux loop (Blilou *et al.*, 2005; Wiśniewska *et al.*, 2006; Rahman *et al.*, 2010). In the present study, the expression of AUX1::AUX1–YFP, PIN2::PIN2–GFP and PIN3::PIN3–GFP was strongly downregulated by a high-P supply but upregulated by a low-P supply, and thus influenced an auxin reflux loop and altered cell division and root deviation. These effects were greater under low Mg than under high Mg, implying that P together with Mg interactively changed auxin redistribution through AUX1, PIN2, and PIN3. The asymmetric distribution of auxin in roots under LMgHP was caused mainly by basipetal transport through the auxin influx AUX1 and auxin efflux carrier PIN2 (Fig. 4). All the findings suggest that mutations within AUX1 and PIN2 caused the root deviation, and that local auxin transport is of primary importance to root skewing on agar. This conclusion was further supported by the physiological results of the application of either 1-NOA or NPA, although the direction of deviation was not identical among the treatments. Moreover, the morphology of root growth of auxin transporter mutants *aux1-22* and *eir1-1* (a loss-of-function mutant for *PIN2*) corresponded to the results of 1-NOA or NPA.

This study also showed that P and Mg interactively regulated auxin redistribution by the transcriptional level of *AUX1*. The enhanced expression of genes *LAX1*, *LAX2*, and *LAX3* under LMgHP treatment but the suppressed expression of these genes under HMgLP treatment (Fig. 7) further confirmed that auxin redistribution controlled by *AUX1* and *LAX* genes was positively involved in regulation of root growth under high P plus low Mg. However, the expression of genes *PIN2* and *PIN3* under high P revealed that the decrease in PIN2 and PIN3 protein levels was not completely mediated at the transcriptional or mRNA stability level (Fig. 4). The strong PIN–GFP signal found under HMgHP suggested that, in addition to *PIN* mRNA abundance, other mechanisms are also involved in the PIN accumulation and auxin-transport capacities. The asymmetrical localizations of AUX1::AUX1–YFP and PIN2::PIN2–GFP at the epidermis and cortex of the meristem (Fig. 4) presumably divert auxin flow to one side in the root meristem, where auxin accumulates and stimulates growth to promote the deviation.

Galinha *et al.* (2007) reported that high levels of *PLT* genes promotes stem-cell identity and maintenance, while low levels enhanced the mitotic activity of the stem-cell niche. Moreover, the expression of *PLT1* and *PLT2* is regulated by auxin at the transcriptional level and depends on ARFs (Aida *et al.*, 2004). The increased expression of *ARF7* and *PLT1*/*PLT2* under LMgHP in this study (Fig. 7) suggests that ARFs and PLTs maintain not only stem-cell identity but also cell proliferation in the proximal meristem as a modulated pattern of auxin redistribution. Otherwise, the expression of *ARR7* and *PLT1*/*PLT2* genes under HMgLP (Fig. 7) is likely to restrain auxin signalling in the basal-cell lineage to affect stem-cell identity and morphology, and eventually to arrest root development. Importantly, the auxin/*PLT* and *SCR/SHR* pathway plays a crucial role in the specification and maintenance of the root stem-cell niche (Sabatini *et al.*, 2003; Dinneny and Benfey, 2008; Kornet and Scheres, 2009). In addition, *LOW PHOSPHATE ROOT1* (*LPR1*) and its close paralogue *LOW PHOSPHATE ROOT2* (*LPR2*) positively regulate the growth of primary roots under P-limited conditions by influencing the activity and distribution of a hormone-like compound (Sánchez-Calderón *et al.* 2005; Svistoonoff *et al.*, 2007). Transcript analysis further confirms that primary root elongation and directional alteration respond to P with a Mg supply by the auxin/*PLT* and *SCR/SHR* pathway that plays a role in the specification and maintenance of the root stem-cell niche. The relationship between the *SHR/SCR* pathway, *PLT1*/*PLT2*, and auxin signalling mechanisms in root development is complex. In this scenario, P and Mg may play synergistic roles in modulating root development at early stages.

Finally, our study suggested that P and Mg interactively affect root elongation and growth direction by a pathway that is largely dependent on the signalling function of *AUX1* and of *PIN2* and *PIN3*, which leads to accelerated cell expansion and root deviation curvature through the redistribution and accumulation of auxin. This information provides new insights into the development of a root system necessary for plant adaptation to an unbalanced supply of nutrients or heterogeneous distribution in the soil, which commonly occurs under the current situation of declining nutrient reserves and food security.

## Supplementary data

Supplementary data can be found at *JXB* online.

Supplementary Table. S1. The nutrient solution composition of the phosphate- magnesium-addition solutions.

Supplementary Fig. S1. Elongation and directional growth and length of primary root grown for 7 d and for 14 d of wild-type *Arabidopsis* seedlings vertically grown in agar medium with different concentrations of P and Mg.

Supplementary Fig. S2. Elongation and directional growth and length of primary root grown for 7 d and for 14 d of wild-type *Arabidopsis* seedlings vertically grown in agar medium with different concentrations of P and Mg as in Fig. 1 with replacement of MgSO_4_ by MgCl_2_.

Supplementary Fig. S3. The gravity response of the primary root to P and Mg in the agar medium.

Supplementary Fig. S4. *Arabidopsis* seeds exposed to a split-plate experiment where the top/bottom halves of plates were supplied with three combinations of Mg and P levels.

Supplementary Fig. S5. Confocal fluorescence micrographs of PI-stained primary root tips of wild-type *Arabidopsis* seedlings grown for 7 d with the same treatments as in Fig. 1.

Supplementary Fig. S6. *Arabidopsis* marker line SHR::SHR–GFP grown vertically with the same P and Mg treatments as in Fig. 1.

Supplementary Data

## Abbreviations:

1-NOA: 1-naphthoxyacetic acid
GFP: green fluorescent protein
GUS: β-glucuronidase
NPA: N-1-naphthylphthalamic acid
PI: propidium iodide
QC: quiescent centre
YFP: yellow fluorescent protein.
