# Supplementary material for: Phosphorus and magnesium interactively modulate the elongation and directional growth of primary roots in Arabidopsis thaliana (L.) Heynh
Source: J Exp Bot. 2015 Apr 28;66(13):3841–54. doi: 10.1093/jxb/erv181 (PMC4473981; doi:10.1093/jxb/erv181)
Supplement: Supplementary Data [file supp_66_13_3841__index.html]

Phosphorus and magnesium interactively modulate the elongation and directional growth of primary roots in Arabidopsis thaliana (L.) Heynh — Phosphorus and magnesium interactively modulate the elongation and directional growth of primary roots in Arabidopsis thaliana (L.) Heynh — Phosphorus and magnesium interactively modulate the elongation and directional growth of primary roots in Arabidopsis thaliana (L.) Heynh — Supplementary Data 

# Phosphorus and magnesium interactively modulate the elongation and directional growth of primary roots in *Arabidopsis thaliana* (L.) Heynh

## Supplementary Data

Data files

**Files in this Data Supplement:**

- Supplementary Data - Supplementary Data
