# Supplementary material for: Phosphorus and magnesium interactively modulate the elongation and directional growth of primary roots in Arabidopsis thaliana (L.) Heynh
Source: J Exp Bot. 2015 Apr 28;66(13):3841–54. doi: 10.1093/jxb/erv181 (PMC4473981; doi:10.1093/jxb/erv181)
Supplement: Supplementary Data [file supp_erv181_jexbot144691_file001.pdf]

Supplementary Material

Phosphorus and magnesium interactively modulate elongation and directional growth of primary roots by promoting auxin biosynthesis and redistribution in *Arabidopsis thaliana* (L.) Heynh

Yaofang Niu, Gulei Jin, Xin Li, Caixian Tang, Yongsong Zhang, Yongchao Liang, Jingquan Yu

Table. S1 The nutrient solution composition of the phosphate- magnesium-addition solutions.

Treatments/Concentration (μM)	NaH_2PO_4	$MgSO_4$	Na_2SO_4	KNO_3	$(NH_4)_2SO_4$	$CaCl_2$
LMgLP($MgSO_4$)	0.5	1	1500	1500	250	1000
CMgLP($MgSO_4$)	0.5	1000	1500	1500	250	1000
HMgLP($MgSO_4$)	0.5	10000	1500	1500	250	1000
LMgCP($MgSO_4$)	500	1	1250	1500	250	1000
CMgCP($MgSO_4$)	500	1000	1250	1500	250	1000
HMgCP($MgSO_4$)	500	10000	1250	1500	250	1000
LMgHP($MgSO_4$)	3000	1	0	1500	250	1000
CMgHP($MgSO_4$)	3000	1000	0	1500	250	1000
HMgHP($MgSO_4$)	3000	10000	0	1500	250	1000
Treatments/Concentration (μM)	NaH_2PO_4	$MgCl_2$	Na_2SO_4	KNO_3	$(NH_4)_2SO_4$	$CaCl_2$
LMgLP($MgCl_2$)	0.5	1	1500	1500	250	1000
CMgLP($MgCl_2$)	0.5	1000	1500	1500	250	1000
HMgLP($MgCl_2$)	0.5	1000	1500	1500	250	1000
LMgCP($MgCl_2$)	500	1	1250	1500	250	1000
CMgCP($MgCl_2$)	500	1000	1250	1500	250	1000
HMgCP($MgCl_2$)	500	10000	1250	1500	250	1000
LMgHP($MgCl_2$)	3000	1	0	1500	250	1000
CMgHP($MgCl_2$)	3000	1000	0	1500	250	1000
HMgHP($MgCl_2$)	3000	10000	0	1500	250	1000

Note: The source of all other microelement materials was the same as in the 'Materials and methods'.

Figure S1

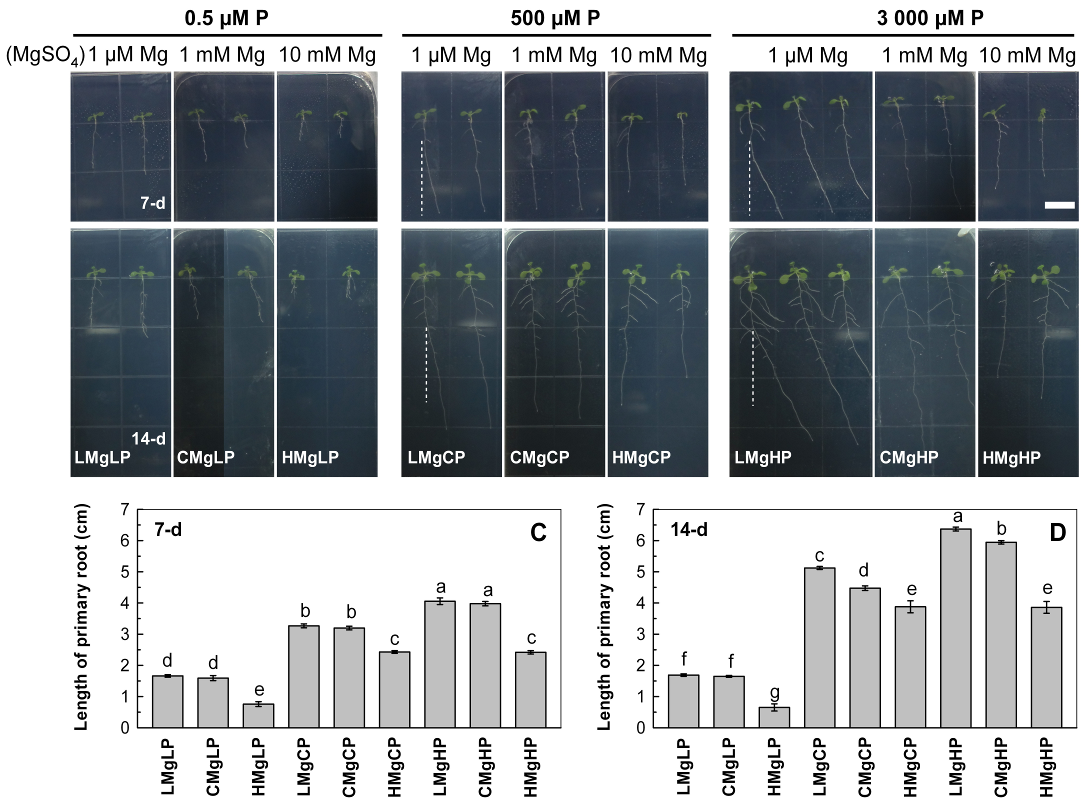

Figure S1. Elongation and directional growth (A, B) and length of primary root grown for 7 d (C) and for 14 d (D) of wild-type *Arabidopsis* seedlings vertically grown in the agar medium with different concentrations of P and Mg. LMgLP, the low Mg and low P medium containing 1 µM Mg^{2+} and 0.5 µM $H_2PO_4^-$; CMgLP, normal Mg and low P medium containing 1000 µM Mg^{2+} and 0.5 µM $H_2PO_4^-$; HMgLP, high Mg and low P medium containing 10000 µM Mg^{2+} and 0.5 µM $H_2PO_4^-$; LMgCP, the low Mg and normal P medium containing 1 µM Mg^{2+} and 500 µM $H_2PO_4^-$; CMgCP, the normal Mg and normal P medium containing 1000 µM Mg^{2+} and 500 µM $H_2PO_4^-$; HMgCP, the high Mg and normal P medium containing 10000 µM Mg^{2+} and 500 µM $H_2PO_4^-$; LMgHP, low Mg and high P medium containing 1 µM Mg^{2+} and 3000 µM $H_2PO_4^-$; CMgHP, the normal Mg and high P medium containing 1000 µM Mg^{2+} and 3000 µM $H_2PO_4^-$; HMgHP, high Mg and high P medium containing 10000 µM Mg^{2+} and 3000 µM $H_2PO_4^-$. Representative seedlings from each treatment were photographed 7 d and 14 d after germination. Data are means ± SD (n = 10). Means followed by a same letter are not significantly different at $P \leq 0.05$. Bars=1 cm.

Figure S2

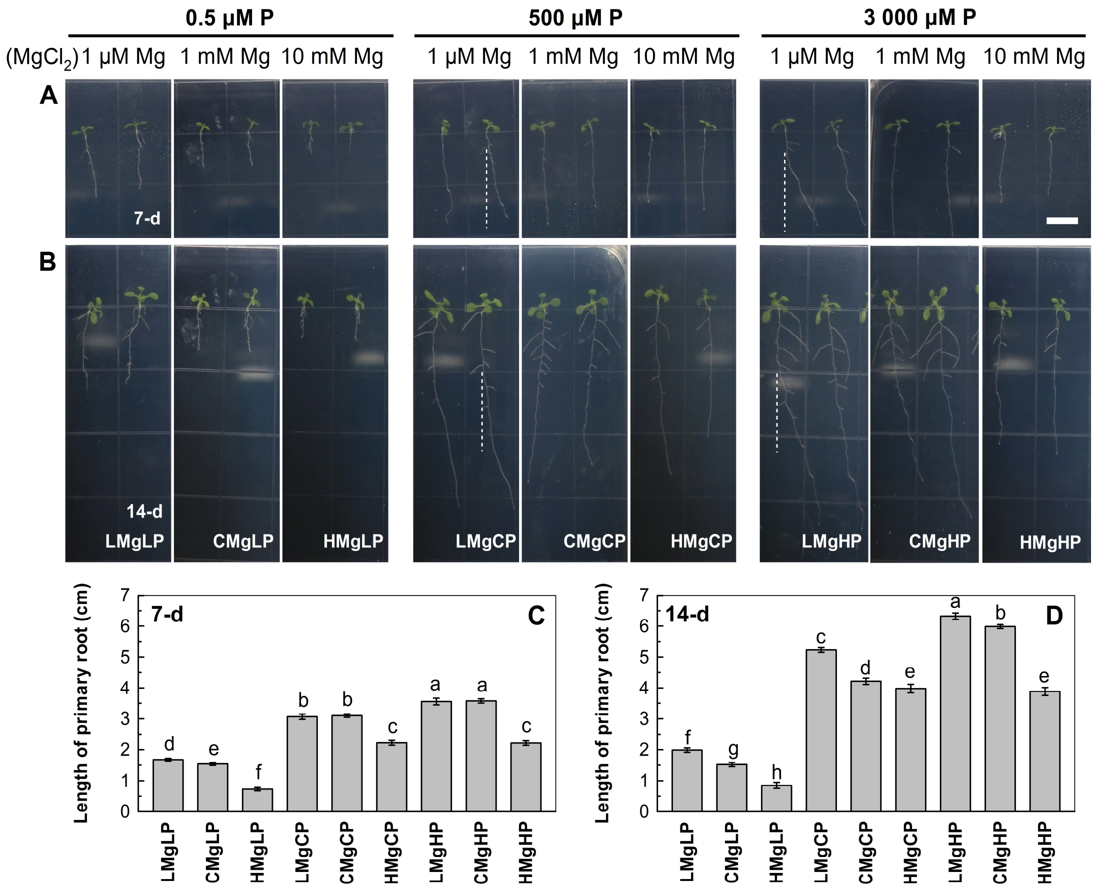

Figure S2. Elongation and directional growth (A, B) and length of primary root grown for 7 d (C) and for 14 d (D) of wild-type *Arabidopsis* seedlings vertically grown in the agar medium with different concentrations of P and Mg as Figure 1 that replacement of $MgSO_4$ with $MgCl_2$. Representative seedlings from each treatment were photographed 7 d and 14 d after germination. Data are means ± SD (n = 10). Means followed by a same letter are not significantly different at $P \leq 0.05$. Bars=1 cm.

Figure S3

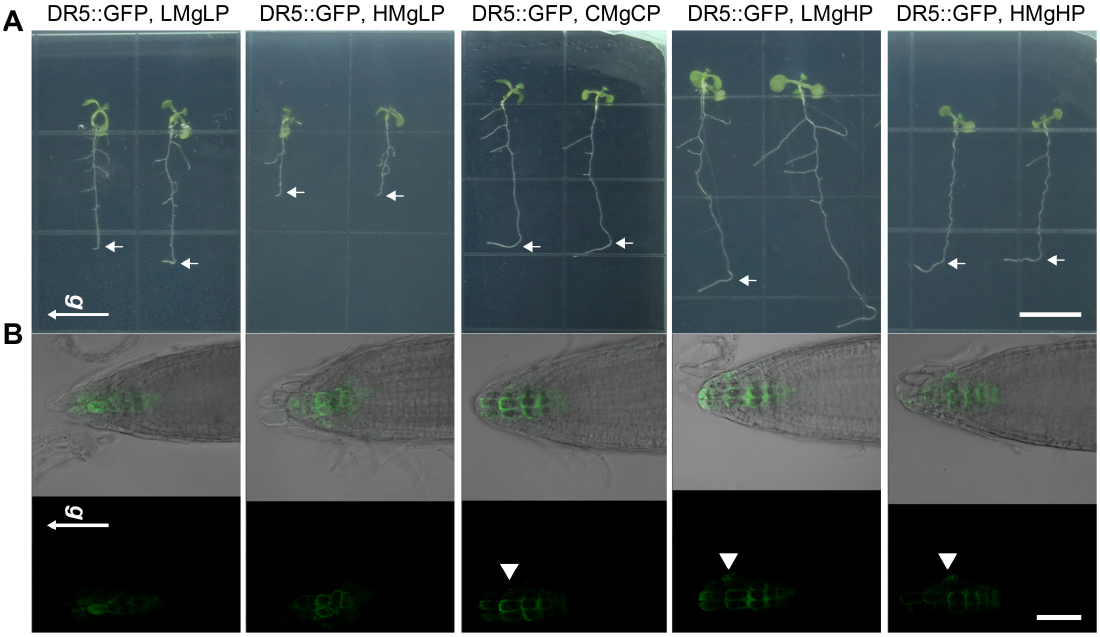

Figure S3. The gravity response of the primary root to P and Mg in the agar medium. Six day after transferred to the split-plate medium, DR5::GFP plant seedlings were rotated 90 degree for 24 h and the images were taken (A, B). GFP fluorescence of root tips in response to gravistimulation (B). Seedlings were cultured as described in Figure 1. Bars = 1 cm for A, bars = 50 µm for B.

Figure S4

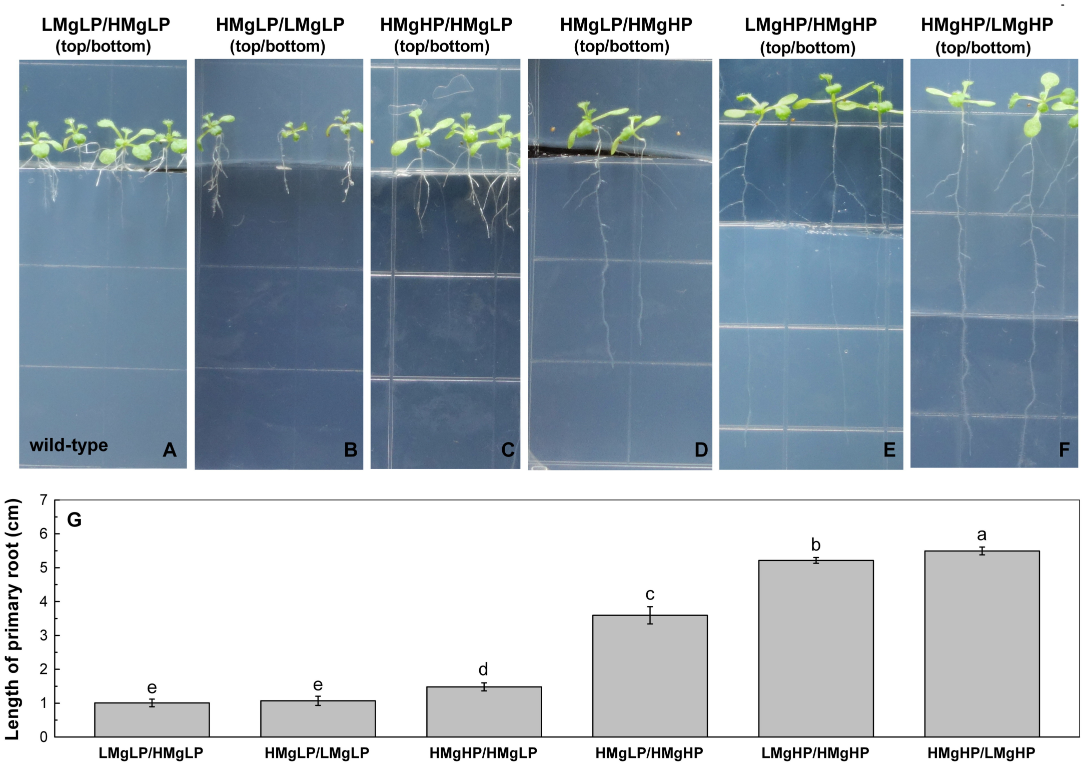

Figure S4. Wild-type *Arabidopsis* seedlings exposed to a split-plate experiment where the top/bottom halves of plates supplied with three combinations of Mg and P levels: LMgLP/HMgLP (A), HMgLP/LMgLP (B), HMgHP/HMgLP (C), HMgLP/HMgHP (D), LMgHP/HMgHP (E), HMgLP/HMgHP (F) and length of primary root (G). Data are means ± SD (n = 6). Means followed by a same letter are not significantly different at $P \leq 0.05$.

Figure S5

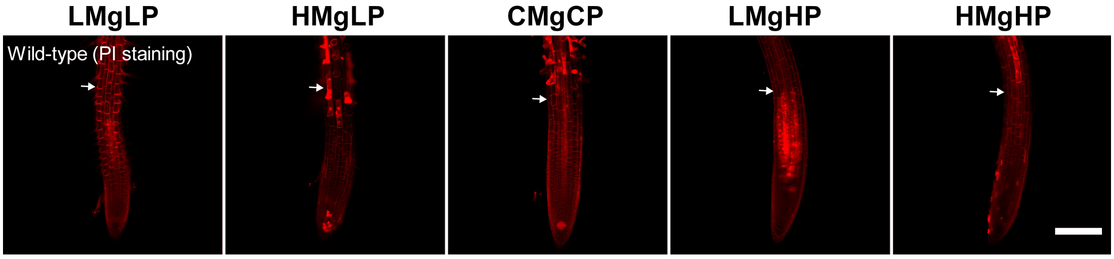

Figure S5. Confocal fluorescence micrographs of PI-stained primary root tips of wild-type *Arabidopsis* grown for 7 d in the same treatments as in Figure 1. Images were collected using a Zeiss 780 confocal microscope with a 20× magnification. Images are representative of 10 plants in three two experiments. Arrowheads indicate the approximate position where cells in elongation zone or root hair zone noticeably. The elongation zone is the distance from the root tip cells of equal distance cell Bar = 100 μm applies to all photographs.

Figure S6

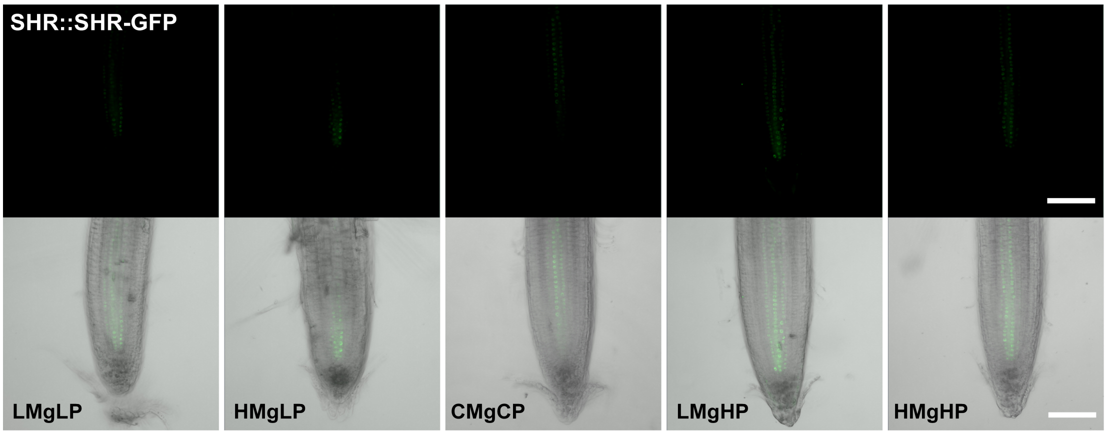

Figure S6. *Arabidopsis* marker lines SHR::SHR-GFP were vertically grown in the same P and Mg treatments as Figure 1. GFP fluorescence of root tips was recorded by using a Zeiss 780 confocal microscope. Scale bar = 50 μm applies to all photographs.
